# Hybrid Polymer Salogels for Reversible Entrapment of Salt-Hydrate-Based Thermal Energy Storage Materials

**DOI:** 10.1021/acsaenm.3c00522

**Published:** 2023-12-08

**Authors:** Kartik Kumar Rajagopalan, Sebastian Haney, Patrick J. Shamberger, Svetlana A. Sukhishvili

**Affiliations:** † Department of Materials Science & Engineering, Texas A&M University, College Station, Texas 77843, United States; ‡ Department of Materials Science & Engineering, University of California, Berkeley, California 94720, United States

**Keywords:** phase change materials, inorganic salt hydrates, polyacrylamide, temperature reversible, salogel, gelation temperature

## Abstract

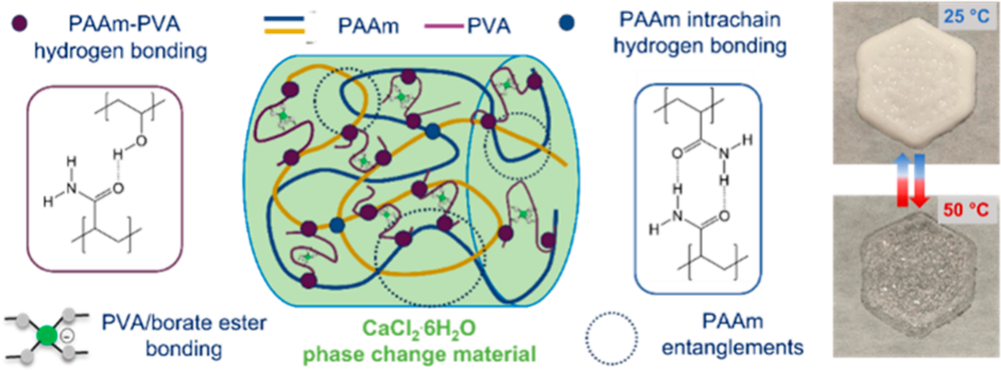

One of the challenges preventing wide use of inorganic salt hydrate phase change materials (PCMs) is their low viscosity above their melting point, leading to leakage, phase segregation, and separation from heat exchanger surfaces in thermal management applications. The development of a broad strategy for using polymers that provide tunable, temperature-reversible shape stabilization of a variety of salt hydrates by using the lowest possible polymer concentrations is hindered by differences in solubility and gelation behavior of polymers with change in the type of ion. This work addressed the challenge of creating robust, temperature-responsive shape-stabilizing polymer gels (i.e., salogels) using a low cost PCM, calcium chloride hexahydrate (CaCl_2_·6H_2_O, CCH). Due to the extremely high (9 M) concentration of chloride ions and the tendency to salting-out polymer chains, the previous strategy of using single-polymer salogels was not successful. Thus, this work introduced a strategy of using two polymers, poly(vinyl alcohol) and ultrahigh molecular weight polyacrylamide (PVA and PAAm, respectively), along with borax as a cross-linker to achieve temperature-reversible, shape-stable salogels. This system resulted in robust salogels whose gel-to-sol transition temperature (*T*_gel_) was tunable within an application-relevant range of gelation temperature (30–80 °C). This behavior was enabled by a synergistic combination of dynamic covalent cross-links between PVA units and entanglements of PAAm chains which were combined into a single hybrid network. The hybrid salogels had <5 wt % polymer content, maintaining ∼95% of the heat of fusion of the pure PCM. Importantly, the noncovalent nature of gelation supported thermo-reversibility of gelation, shape stability, and retention of thermal properties over 50 melting/crystallization cycles.

## Introduction

Energy and environmental crises arising from rapid economic growth and over-reliance on fossil fuels have led to a situation of dire need for both alternate energy sources and storage technologies to meet the gap between demand and supply.^1,2^ Thermal energy storage technologies using phase change materials (PCMs), which can store and release large amounts of energy in the form of latent heat, are becoming important in solar thermal energy applications, industrial waste heat recovery, and building thermal regulation.^1−3^ Incorporation of PCM materials in building walls and thermal energy storage modules allows heat capture and cooling during the day while energy released at night can be used for heating over many cycles.^2^ Among the types of materials used as PCMs in building applications - organic^4−7^ and inorganic,^5,6^ inorganic salt hydrates are gaining traction because of their high volumetric latent heat storage capability, low cost, high thermal conductivity, and nonflammability in comparison to their organic counterparts.^2,3,5,6,8^ Widespread use of salt hydrates has been limited, however, by lack of shape stability of these PCMs in the liquid state due to their low viscosity that makes them susceptible to leakage from thermal energy storage modules.^2,3,9^

Shape stabilization strategies for salt hydrates PCMs can be broadly classified into three categories: encapsulation, impregnation in a porous matrix, and entrapment in organic three-dimensional network structures.^9^ In addition, polymer thickeners, which increase the viscosity of salt hydrates, are often added to molten PCMs to prevent phase segregation. However, the relatively large amounts (5%–25% by weight) of the added thickeners reduce the PCM content, decreasing the heat storage capability. Moreover, polymer thickeners do not provide shape stabilization or prevent leakage of molten PCMs (Figure S1).^10−23^ In contrast, covalently crosslinked polymer gels, which also often require large polymer amounts (5%–60% by weight), provide shape stabilization (Figure S1).^24−40^ However, the permanent nature of the crosslinks renders these materials irreversible, making filling and removal from thermal energy storage modules impossible. In contrast to these previous strategies, our group introduced temperature-responsive polymer salogels (polymer gels in inorganic salt hydrates) based on hydrogen bonding polymers such as poly(vinyl alcohol) (PVA), for reversible shape stabilization of liquid salt hydrates.^41,42^ The on-demand reversibility of gelation by heating above a gel-to-sol transition temperature (*T*_gel_) allows filling and removal of the salogels from thermal energy storage devices, whereas gelation below *T*_gel_ provides shape stabilization above the melting temperature of the salt hydrate (*T*_m_). Note that such on-demand reversibility is not possible with either encapsulation or impregnation methods because both types of matrices lack temperature-triggered reversibility. Our prior work demonstrated that hydrogen bonding polymers form gels in liquid inorganic salt hydrates because of the unique behavior of salt hydrate solvents where the high ionic concentration and scarcity of water result in incomplete saturation of hydration shell of ions.^41,42^ This causes dehydration of polymer chains due to competition between the ions and polymers for the water in the salt hydrate, inducing polymer–polymer and polymer–ion interactions that facilitate gelation of PVA at relatively low polymer concentrations (5–15 wt % in LiNO_3_·3H_2_O (LNH) and Ca(NO_3_)_2_·4H_2_O (CNH)),^41,42^ at which PVA gels do not form in aqueous solutions. The developed salogels provided shape stabilization of the nitrate salt hydrates above the PCM melting temperature but could still reversibly revert to a liquid by temperature-induced dissociation of the hydrogen bonded polymer network at a gel-to-sol transition temperature, *T*_gel_. The addition of hydrogen bonding and dynamic covalent crosslinkers allowed further strengthening of the salogel and tunability of *T*_gel_ over a wide temperature range by varying the crosslinker concentration.^43,44^ These strategies allowed us to create salogel systems for nitrate salt hydrates with high PCM loading (>90 wt %) enabling retention of thermal energy storage capability while simultaneously providing efficient shape stabilization and on-demand reversibility of gelation to enable filling and removal. A comparison of salogels with thickeners and covalent networks reported in literature based on PCM amounts is provided in Figure S1.

In this work, we explore salogel systems in a different, chloride-based PCM: calcium chloride hexahydrate (CaCl_2_·6H_2_O, CCH). CCH has low toxicity and is economical compared to lithium-based salt hydrates as it is generated as a waste byproduct in chemical processes such as soda ash production, and is also naturally abundant.^8,45,46^ In addition, a melting temperature of 29 °C and heat of fusion of 190 J/g make this salt hydrate ideal for thermal energy storage applications in waste heat recovery, textiles, and buildings.^8,40,46^ Shape stabilization of CCH by impregnation in a porous matrix^47−50^ or encapsulation^51,52^ has been demonstrated, but these approaches lack temperature-triggered reversibility and also result in a significantly reduced heat of fusion compared to that of neat CCH. Similarly, the use of polymer thickeners for CCH,^21,23^ or permanently cross-linked networks for the CCH-MgCl_2_·6H_2_O eutectic,^40^ lacks the desired shape stabilization and temperature reversibility. Here, we address the challenge of thermo-reversible shape stabilization of CCH by introducing the hybrid salogel strategy. The novel strategy was necessary as the switch from nitrate (CNH) to chloride (CCH) salt hydrate resulted in drastic changes in the gelation behavior of PVA so that strong and temperature-responsive salogels could no longer be prepared at low polymer concentrations (∼3–4 wt %) using our previous strategy involving a single polymer network based on PVA/borax dynamic covalent bonding.^44^ Hence, we introduce a strategy of making a hybrid salogel system where the polymer networks are formed at low total polymer concentrations (<5 wt %) by the synergistic effect of intermolecular hydrogen bonding between PVA and ultrahigh molecular weight polyacrylamide (PAAm, 6000 kDa). The PVA and PAAm are further reinforced by boronate ester bonds and entanglements, respectively. The resulting hybrid network consisting of boronate ester bonds and entanglements works synergistically in a single network to provide shape stabilization and leakage prevention of CCH. The thermo-reversible nature of all these interactions supports a tunable temperature response and retention of thermal properties of CCH in the salogel even after repeated thermal cycling.

## Materials and Methods

### Materials

PVA (molecular weight 90 kDa, 98% hydrolyzed) and sodium tetraborate decahydrate (borax) (ACS, 99.5%–105.0%) were purchased from Alfa Aesar and used without modification. Calcium nitrate tetrahydrate (ACS, 99.0%–103.0%) and anhydrous calcium chloride (96%) were purchased from Alfa Aesar and used without modification. Deuterium oxide (D_2_O) with 99.9 atom % and polyacrylamide (6000 and 150 kDa) were purchased from Sigma-Aldrich and used as received. Calcium chloride hexahydrate (CCH), CaCl_2_·6H_2_O (99%, calculated based on dry substance), was obtained from Sigma-Aldrich and was used as received.

### Preparation of Salogels

Salogels were prepared by adding a solid polymer (PVA and/or PAAm) into liquid chloride salt hydrate (CCH) with gentle stirring and heating at 80 °C on a hot plate in a sealed vial until the polymers dissolved. Both PVA and PAAm dissolved in CCH in about 24 h of mixing at 80 °C. The polymer–CCH mixture was removed from the hot plate and placed in an oven at 80 °C overnight to remove bubbles and obtain a homogeneous solution which was cooled to room temperature to induce gelation to form borax-free salogels. Borax containing salogels were prepared by adding borax (amount calculated as mol % of borax to PVA hydroxyl groups) to the polymer–CCH mixture followed by heating the mixture to 85 °C for 24 h while stirring to facilitate the dissolution of borax. Once the borax dissolved, the polymer–borax–CCH mixture was removed from the hot plate and placed in an oven at 85 °C overnight to remove air bubbles. The homogeneous mixture obtained was then cooled to room temperature to induce gelation. Salogel preparation flowchart is shown in Scheme 1 in the Supporting Information. Because of the known supercooling effect that is significant in the case of CCH,^40^ no crystallization occurred in the salogel systems at room temperature. However, crystallization occurred at refrigeration temperature of 4 °C in a few hours in contrast to CNH salogels reported in our previous work which required freezing temperatures for several hours.^44^

### Deuteration of CNH and CCH for ATR-FTIR Studies

The use of deuterium oxide (D_2_O) in place of H_2_O in salogels enabled distinguishing the −OH stretching band of water from the salt hydrates and observing the −NH band in PAAm (3200–3350 cm^–1^). To prepare the deuterated analogue of CCH, i.e., CCD, anhydrous calcium chloride was mixed with the stoichiometric amount of D_2_O (moles of water per mole of anhydrous salt, *n* = 6) to obtain CCD. Analysis by attenuated total reflection Fourier transform infrared spectroscopy (ATR-FTIR) showed the disappearance of the −OH peak (3000–3800 cm^–1^) and the appearance of the −OD peak (2100–2800 cm^–1^) after the completion of the above procedure, thus confirming the successful deuteration of CNH (Figure S2). Deuterated CNH (CND) was prepared using the procedure described in our previous work.^44^ Briefly, CNH was dried in a vacuum oven at 135 °C to remove the water of crystallization to obtain anhydrous calcium nitrate. Anhydrous calcium nitrate was then mixed with a stoichiometric amount of water (moles of water per mole of anhydrous salt, *n* = 4) to prepare CND with successful deuteration confirmed from ATR-FTIR (Figure S2). To study the effect of water concentration on gelation behavior of PVA in the chloride (CC*n*D) and nitrate (CN*n*D) salt hydrates, the number of moles of water per mole of anhydrous salt was varied between 4 and 12 by adding an appropriate amount of D_2_O to the anhydrous calcium chloride and calcium nitrate salts.

### Materials Characterization

#### ATR-FTIR

ATR-FTIR measurements were performed on a Bruker Tensor II spectrometer equipped with a mercury cadmium telluride (MCT) detector and a single-reflection diamond ATR attachment. Spectra were collected in the range of 400–4000 cm^–1^ at 4 cm^–1^ resolutions using 64 repetitious scans. Each measurement was performed with ∼10 μL of the sample drop-cast onto the ATR diamond crystal.

#### Dynamic Light Scattering (DLS)

Dynamic light scattering (DLS) experiments were conducted in a custom-made instrument using a laser wavelength of 532 nm and a scattering angle of 90°. The samples were filtered through a 0.45 μm PTFE syringe filter before loading into a 12 mm × 12 mm plastic cuvette and were allowed to equilibrate overnight at room temperature in the cuvette before taking measurements. Measurements were performed at room temperature at a PVA concentration of 0.5 mg/mL in both CNH and CCH.

#### Rheological Measurements

Rheological measurements for salogel samples were performed using a TA Instruments HR2 Discovery Hybrid rheometer equipped with a Peltier stage that enabled temperature control within ±0.5 °C. All measurements were performed using a parallel plate with a 40 mm diameter and a gap of 500 μm. After loading on the rheometer, the salogel samples were initially heated to 80 °C for 5 min to remove any thermal history followed by cooling down to room temperature. The samples were allowed to relax for 5 min at room temperature to ensure that the gel networks formed completely before beginning the experiments. The linear viscoelastic regime (γ_L_) was determined by oscillation amplitude sweep experiments conducted at 25 °C within a strain range of 0.1%–100% at a frequency of 10 rad/s. The oscillation temperature ramp experiments were performed in the linear viscoelastic regime at a frequency of 10 rad/s and 1% strain by heating the sample from 25 to 90 °C. *T*_gel_ was determined from the crossover of storage (*G*′) and loss (*G*′′) moduli. Water evaporation and absorption during the experiment were minimized using a solvent trap.

#### Thermal Analysis

The thermal properties (melting temperatures and heat of fusion) of neat salt hydrate (without polymer) and in salogels were determined by differential scanning calorimetry (DSC) using a TA Instruments Q2000. Measurements were conducted at a 10 °C/min temperature ramp rate from −40 to 80 °C under nitrogen gas purging at a flow rate of 50 mL/min. Hermetic aluminum DSC pans were used for all the samples (neat salt hydrate and salogels) and were prepared in a drybox under an inert gas (nitrogen) environment at a controlled humidity of 20% to ensure contamination from ambient moisture was avoided.

### Thermal Cycling and Demonstration of Shape Stabilization of Salogels

The ability of the hybrid salogels to shape stabilize, prevent leakage of CCH, and preserve its thermal properties during thermal cycling was evaluated by subjecting a salogel sample sealed in a 20 mL vial under nitrogen in a drybox to multiple melting and crystallization cycles. Salogels containing 2 wt % PAAm/2 wt % PVA/10 mol % borax were chosen for these experiments. Melting was achieved by heating the vial to 50 °C until all of the CCH melted. The vial was then allowed to equilibrate to room temperature and then put in the refrigerator to induce crystallization. The DSC scan was performed after 50 cycles by preparing the pan in the controlled humidity, inert environment of the drybox. DSC and rheology experiments were performed after 50 melting–crystallization cycles to test the preservation of thermal and mechanical properties of the salogel. Shape stabilization experiments were performed using hexagon-shaped and alphabet-shaped (“T”, “A”, “M”, and “U”) salogels obtained by crystallizing the as-prepared salogels poured into molds of various shapes after heating above the gel-to-sol transition temperature. Thermal cycling was done by heating the bulk salogels to a temperature (50 °C) above the melting point of CCH (29 °C) while storing the salogel in an airtight container and holding at this temperature until all the CCH melted.

## Results and Discussion

**Figure 1:**
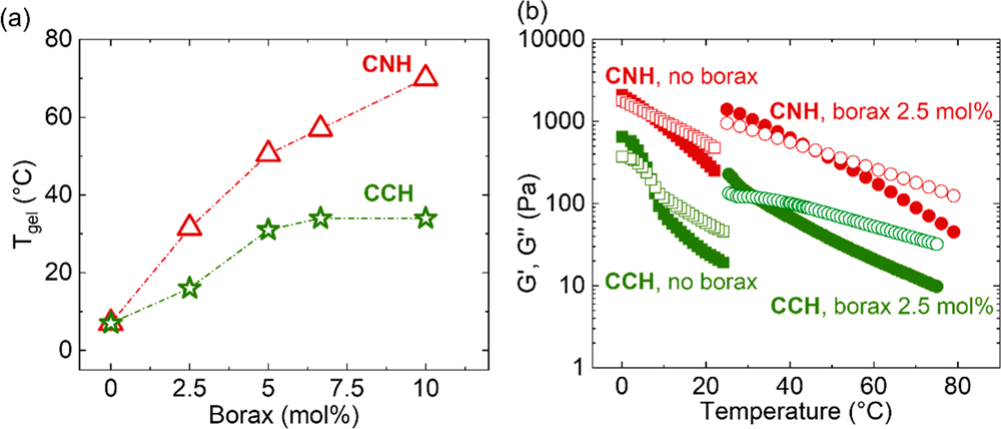
(a) Comparison of *T*_gel_ obtained from temperature sweep rheology experiments for PVA/borax salogels in CNH and CCH. (b) Temperature sweep rheology experiments comparing *G*′ (closed symbols) and *G*″ (open symbols) as a function of temperature for PVA and PVA/borax salogels in CCH and CNH. Polymer concentration was 3 wt %.

In our previous work, we have shown that gelation of PVA in nitrate salt hydrates (CNH and LNH) occurs readily due to the dehydration of polymer chains in the water-scarce environment inducing polymer–polymer and polymer–ion interactions.^41,42,44^ Here, we focused on a chloride-based PCM (CCH) and first compared the gelation behavior of PVA and the strength of the salogels between a nitrate-based salt hydrate (CNH) and CCH. Figure 1 and Figure S3 compare the *T*_gel_, and the temperature dependence of *G*′ and *G*″ for PVA and PVA/borax salogels in liquid nitrate and chloride salt hydrates. For the same borax concentration, the salogels formed in CNH had higher *T*_gel_ which could be tuned over a broader temperature range in CNH (7–75 °C) compared to CCH (7–35 °C) (Figure 1(a)). Comparison of temperature sweep plots showed that the gels in CCH were weaker compared with the gels in CNH (Figure 1(b) and Figure S3).

**Figure 2:**
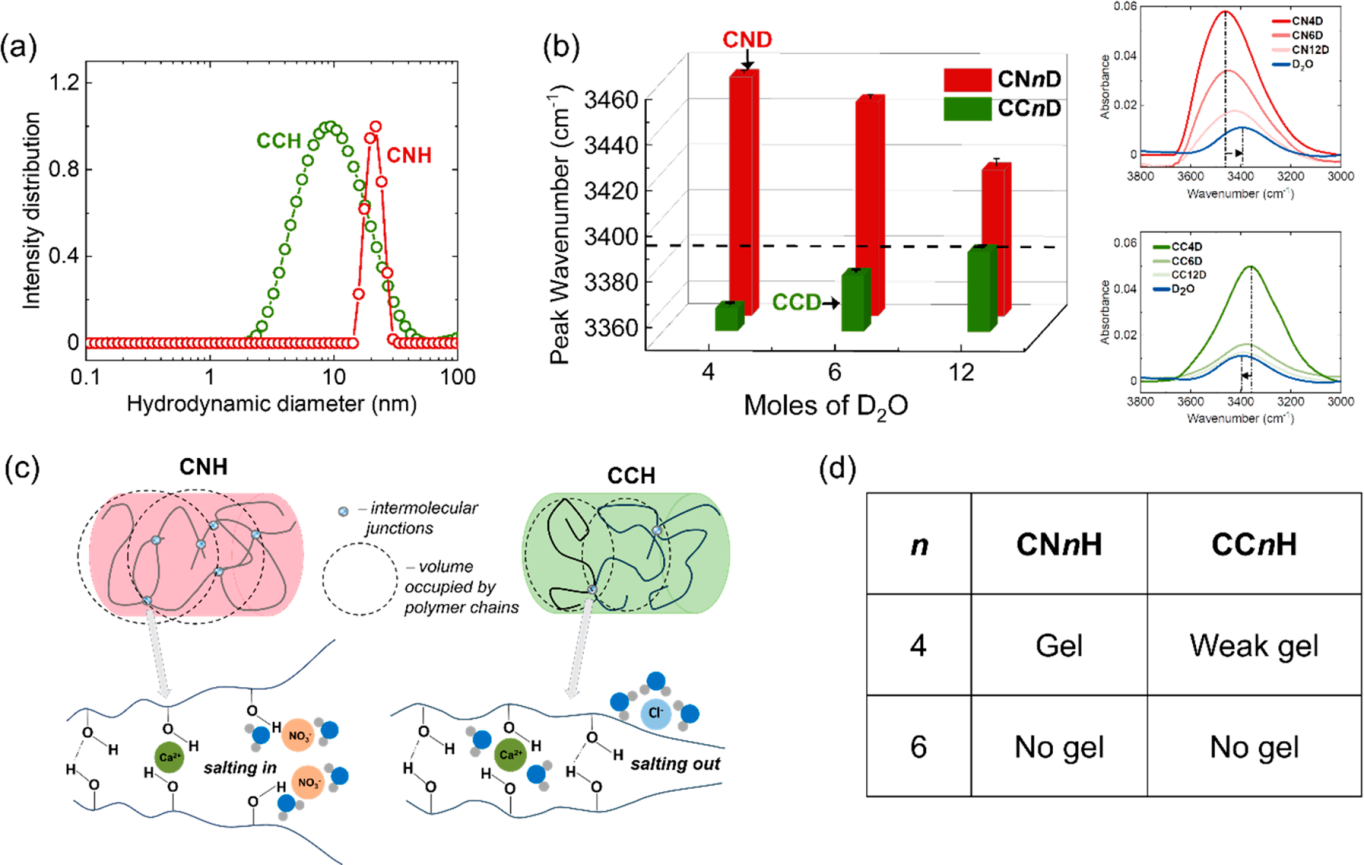
(a) DLS experiments comparing the hydrodynamic diameter of PVA chains in CNH and CCH at 23 °C for a PVA concentration of 0.5 mg/mL. (b) Peak wavenumber of PVA hydroxyl group as a function of water concentration (*n*) in deuterated calcium nitrate (CN*n*D) and calcium chloride (CC*n*D) obtained from ATR-FTIR. Dotted line shows the peak wavenumber in bulk water (D_2_O). The actual salt hydrate PCMs (CN4D and CC6D) are indicated by arrows. Inset shows the spectra of CND (top) and CCD (bottom). (c) Schematics showing the gelation mechanism of PVA in the CNH and CCH. (d) Table indicating at which water concentrations gelation occurs in CNH and CCH at room temperature. PVA concentration was 5 wt % for FTIR and gelation experiments.

To understand the differences in performance of the salogels formed in different salt hydrates, we aimed to study the behavior of polymer chains by DLS and explore the mechanism of gelation of PVA using ATR-FTIR. DLS experiments revealed differences in the hydrodynamic sizes of PVA chains in the liquid CNH and CCH salt hydrates indicating expansion of PVA chains in nitrate-based and more collapsed chains in chloride-based salt hydrate (30 and 9 nm, respectively, Figure 2(a)). To explore the contribution of polymer chain hydration in this behavior, FTIR spectroscopy studies were performed with deuterated salt hydrates (CND and CCD) using a procedure similar to the one used in our previous works.^42,44^ Deuteration of salt hydrates allowed us to observe the changes in the characteristic wavenumber of the stretching vibrations of PVA hydroxyl group (ν_OH_^PVA^) without interference from the hydroxyl group stretching vibrations of H_2_O in the salt hydrate (Figure S2). Note that PVA concentration of 5 wt % was used for ATR-FTIR experiments to increase the intensity of the PVA hydroxyl group peak. Since CNH is a tetrahydrate (*n* = 4, where *n* is the number of moles of water per mole of anhydrous salt) and CCH is a hexahydrate (*n* = 6), we varied the water content in the salt hydrates to differentiate between the roles of water and nature of ions in solubility and gelation of PVA. The water content was varied from 4 mol per mole of anhydrous salt (lowest water content at which CNH and CCH form stable salt hydrates) to 12 mol (complete saturation of first hydration shell of cation and anion) in both salt hydrates. FTIR analysis of the ν_OH_^PVA^ wavenumber at matched water content (*n* = 4, 6, or 12) for the nitrate and chloride salt hydrate indicated a strong effect of anion type on polymer behavior (Figure 2(b)). Specifically, compared to bulk water (D_2_O) where the wavenumber of the hydroxyl group of PVA was 3393 cm^–1^, the peak was significantly blue-shifted to 3454 cm^–1^ in CNH and red-shifted to 3360 cm^–1^ in CCH at low water content (*n* = 4 and 6) (Figure 2(b)). The peak shift indicates differences in hydrogen bonding state of the hydroxyl group.^41,42,44^ These differences can be explained using the Hofmeister effect, which rates anions on a scale based on their ability to cause salting-out (kosmotropes) or salting-in (chaotropes) of polymers in aqueous solutions.^53,54^ The chaotropic behavior of the nitrate ions results in increased solubility of PVA which disrupts polymer–polymer hydrogen bonding and swells chains as revealed in the strong blue shift of the ν_OH_^PVA^ (Figure 2(b)) and from DLS (Figure 2(a)). In contrast, the chloride ions, known to be a transition point from chaotropic to kosmotropic behavior in aqueous solutions,^55^ show strong kosmotropic behavior in the water-scarce salt hydrate environment inducing polymer–polymer hydrogen bonding resulting in a red shift (Figure 2(b)) and collapsed polymer chains (Figure 2(a) and (c)). Figure 2(d) and Figure S4 show that PVA (at a concentration of 5 wt %) formed a gel in the nitrate salt at *n* = 4 at room temperature, whereas in the chloride salt no gelation occurred, and viscous solution started to flow after a few seconds at room temperature as determined from a simple vial inversion experiment. Therefore, water scarcity and high ionic concentration in salt hydrates have a strong effect on polymer solubility and gelation behavior. The addition of borax did not result in the formation of a strong gel network capable of shape stabilizing and preventing leakage of CCH in the liquid state (Figure S5). Similar results have been reported for borax complexation with diols in aqueous solutions^56,57^ and hydrogels^58,59^ containing NaCl. The decrease in viscosity^56,57^ and modulus^58,59^ were rationalized by a charge shielding effect of anionic boronate ester cross-links by chloride ions leading to enhanced intrachain crosslinking and polymer chain collapse.

**Figure 3:**
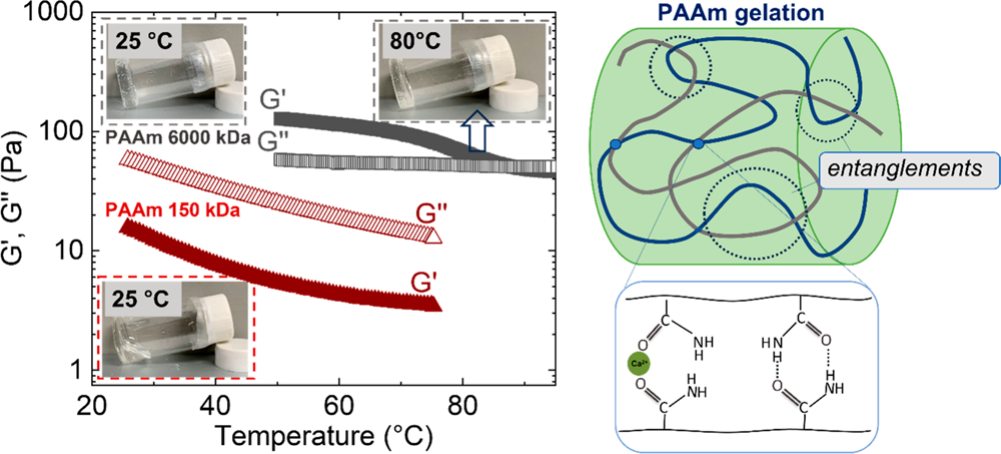
Temperature sweep rheology experiments comparing ultrahigh molecular weight PAAm (6000 kDa) and PAAm (150 kDa) in CCH. Insets show vial inversion experiments showing shape stable gelation with PAAm 6000 kDa (pictures at the top with gray dashed outline) at all temperatures and lack of gelation with PAAm 150 kDa (picture at the bottom with maroon dashed outline). PAAm concentration was 3 wt % for both molecular weights. Temperature sweep was performed at a frequency of 10 rad/s and 1% strain in the viscoelastic regime. Schematics on the right show gelation mechanism for PAAm 6000 kDa.

Having understood that a PVA/borax system is not sufficient to form strong and shape stable gels in CCH at low polymer concentrations, we aimed to overcome this problem by applying a strategy involving the use of an ultrahigh molecular weight polymer which formed a gel network through physical entanglements in addition to hydrogen bonding.^60^ Ultrahigh molecular weight polyacrylamide (PAAm) of molecular weight 6000 kDa was used since PVA is not available commercially in this molecular weight range. Figure 3 shows that PAAm (6000 kDa) formed a physical gel at a low polymer concentration of 3 wt % which was shape stable up to 80 °C but showed reversibility of gelation slightly above this temperature (∼87 °C) due to thermo-responsiveness of entanglements,^61,62^ while no gelation was observed in water (Figure S6). Physical gelation of polymers occurs when the polymer concentration exceeds the overlap concentration (c*) of polymer chains in the solvent.^63^ Overlap concentration of ultrahigh molecular weight PAAm has been reported to be in the range of 0.5–1.6 mg/mL in water for a molecular weight range of 4800–15,000 kDa.^64^ Our concentration of 3 wt % (5 mg/mL) in CCH exceeds that significantly resulting in interchain contacts due to entanglements. Based on the molecular weight of PAAm of 6000 kDa and the known entanglement molecular weight for PAAm of ∼400 kDa in water,^65^ number of entanglements forming a polymer network can be estimated as 15 entanglements per chain. In addition to the high number of entanglements, the PAAm network can be further strengthened by intermolecular hydrogen bonding between the amide groups and polymer–ion interactions, both promoted by the dehydration of polymer chains in the salt hydrate environment (Figure 3 schematics). The dehydration of PAAm chains could be detected from the shifts in the amide peaks (salt hydrate vs aqueous solvents) observed in ATR-FTIR (Figure S7). In contrast, PAAm of lower molecular weight (150 kDa) was incapable of forming entanglements at low concentration and required as high as 10 wt % polymer concentration to form a temperature reversible gel with a reasonably high *T*_gel_ of 42 °C (Figure S8). Within the low range of polymer concentrations (0–4 wt %), PAAms of both molecular weights did not significantly affect the thermal properties (heat of fusion and melting temperature) of the salt hydrate, retaining ∼95% of the heat fusion of CCH and lowering its melting point by only 2 °C for 4 wt % of PAAm concentrations (Figure S9). However, the use of PAAm 6000 kDa for shape stabilization of CCH was hampered by its relatively high *T*_gel_ (>90 °C), which was not readily tunable. This high value of *T*_gel_ makes processing of CCH salogels and filling of heat exchangers difficult, due in part to the relatively high water vapor pressure at this temperature. At the same time, PVA/borax salogels were weak and had a low *T*_gel_ (see Figure 1). Thus, we pursued the strategy of making hybrid PAAm/PVA/borax salogels in order to combine the advantages of the two systems (PVA/borax and PAAm), which provided tunable *T*_gel_ and shape stabilization, respectively.

**Figure 4:**
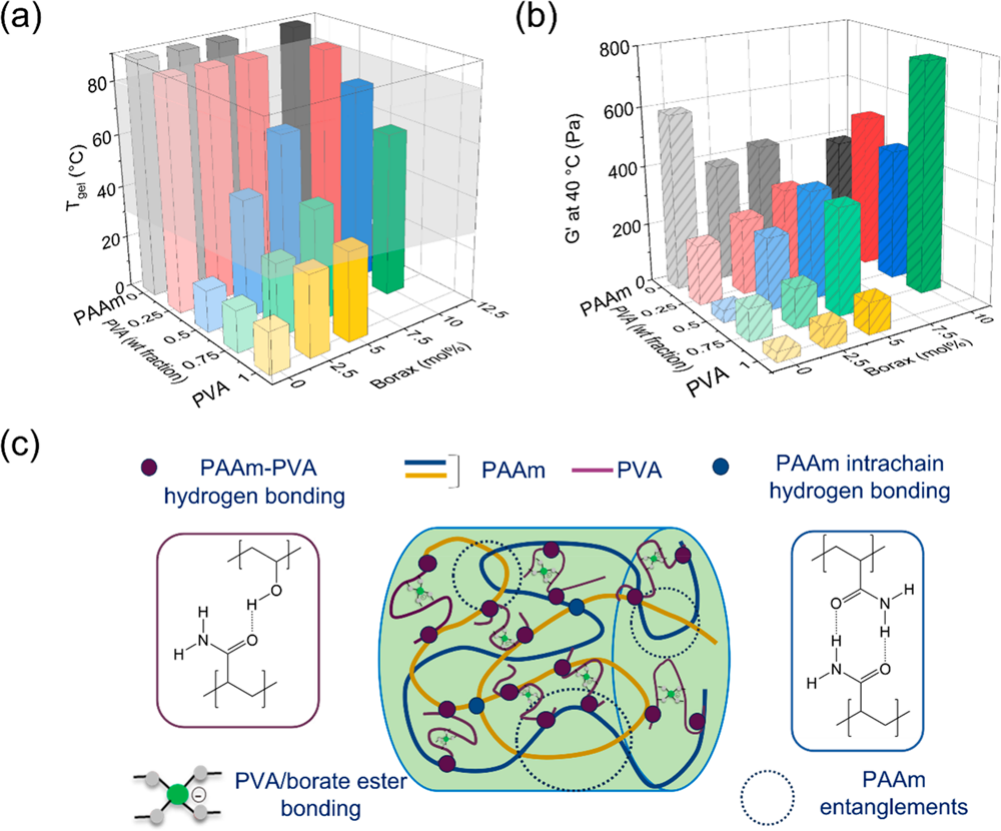
3D bar plots comparing (a) *T*_gel_ obtained from temperature sweep rheology experiments for salogels containing PVA/borax/PAAm in different ratios and (b) *G*′ at 40 °C. All the gels contain 4 wt % total polymer. Note that *T*_gel_ of 100% PAAm salogels in (a) was greater than 90 °C and could not be measured in the rheological experiments. (c) Schematic showing the various interactions involved in the hybrid PVA/borax/PAAm salogels. The gray box in (a) highlights the relevant *T*_gel_ range for the processability of salogels.

Figure 4 summarizes the results from temperature ramp rheological experiments performed with salogels of PVA, PAAm, and their hybrids at different polymer weight ratios with and without the addition of borax. The various salogel systems were compared on the basis of *T*_gel_ and storage modulus (*G*′) (Figure 4) and tan δ values (Figure S10). The *G*′ values were compared at 40 °C (Figure 4(b)), a temperature at which CCH was in its molten state and thus required shape stabilization and leakage prevention. Therefore, a high *G*′ value and a low tan δ value at this temperature were desirable. Note that the salogel systems shown in Figure 4 were at 4 wt % total polymer concentration, as an increase in total polymer concentration improved the salogel strength without sacrificing their temperature responsiveness (Figure S11). Salogels based on a PVA/borax system containing only boronate ester bonds had low *T*_gel_ values and were not even in a gel state at 40 °C(Figure 4(a, b), Figure S12). On the other hand, salogels based on PAAm were stabilized by physical entanglements and intrachain hydrogen bonding and lacked temperature response in the temperature range of interest. Specifically, *T*_gel_ values of PAAm salogels were outside of the reliable temperature range of rheological measurements of salt hydrate materials (i.e., >90 °C) (Figure 4(a)). Increasing borax concentration did not alter the gelation behavior of PAAm or affected tan δ values measured at 40 °C (Figure 4(a) and Figure S10, respectively), while causing a slight decrease (150–200 Pa) in *G*′ at 40 °C which may be due disruption of some intermolecular hydrogen bonds between PAAm by borax (Figure 4(b)). The temperature sweep rheology curves for the PAAm/borax salogels are shown in Figure S12. The data indicate that interactions between PAAm and borax are weak and did not significantly affect the formation of the overall PAAm gel network, as there is no change in *T*_gel_ for the borax concentrations tested.

Combining the two approaches to gelation, entanglements and boronate ester crosslinks, resulted in hybrid salogels with a tunable gelation temperature and mechanical properties. Note that the hybrid salogels showed a single *T*_gel_ in temperature sweep rheology experiments indicating the formation of a single network. The salogels containing larger amounts of PVA (PVA:PAAm 75:25 by weight) had a low *T*_gel_ range (16–62 °C) due to the predominance of PVA–borax bonds which reverse at a low temperature in CCH (Figure 1(a)), while the salogels with larger amounts of PAAm (PVA:PAAm 25:75 by weight) lacked tunability of *T*_gel_ (Figure 4(a, b) and Figure S10). Hybrid salogels containing equal amounts of PVA and PAAm (PVA:PAAm 50:50) with dynamic covalent crosslinks and entanglements, coupled by PAAm-PVA hydrogen bonds within a joint hybrid network, resulted in the desired combination of *T*_gel_ tunability and gel strength. Specifically, *T*_gel_ with tunability over a range of 16–75 °C (Figure 4(a)) by varying borax concentration, and addition of borax in the amount of 10 mol % to molar concentration of PVA units, yielded salogels with 10X higher *G*′ and 2X lower tan δ in comparison to the borax-free counterpart (Figure 4(b) and Figure S10). While the salogel with the equal amount of PVA and PAAm with 10 mol % borax had a slightly lower *G*′ than the 75:25 PVA:PAAm and 25:75 PVA:PAAm, it provided the highest *T*_gel_ and broadest range of tunability of *T*_gel_ while maintaining sufficient strength. The improved *T*_gel_ and mechanical properties are a manifestation of the synergistic effect between the boronate ester cross-links of PVA units and physical entanglements of PAAm that is enabled by the formation of a network of polymer–polymer hydrogen bonding between PVA and PAAm chains (Figure 4(c)) which joined the PVA/borax and PAAm networks together. The synergistic effect of PVA/borax and PAAm is further obvious from comparison of temperature sweep rheology curves of the individual components with the hybrid salogel at matched polymer and borax concentration. While the PVA/borax salogel was not able to shape stabilize CCH, being in the sol state at 40 °C, the PAAm salogel was shape stable but had a *T*_gel_ much higher than 80 °C (Figure S13). In contrast, hybrid salogels were able to maintain strength while exhibiting temperature responsiveness in the relevant range of temperatures (30 to 80 °C). The contribution of each component in the hybrid salogel can be understood from Figure S14 where 2% PAAm formed a gel that lacked temperature response in the relevant range of temperatures, while 2% PVA/borax was temperature responsive, with *T*_gel_ limited to room temperature. When combined, the resulting hybrid network formed by the intermolecular hydrogen bonding between PAAm and PVA enabled a single network with additive effect of entanglements and boronate ester crosslinks resulting in a higher gel strength and *T*_gel_ which is tunable within a wide temperature range. Therefore, the ability of hydrogen bonds, boronate ester crosslinks, and even entanglements to dissociate and break upon heating supports the temperature response is important for practical applications as a means for facile removal of a salogel from a heat exchange module at the end of life of a PCM material.

**Figure 5:**
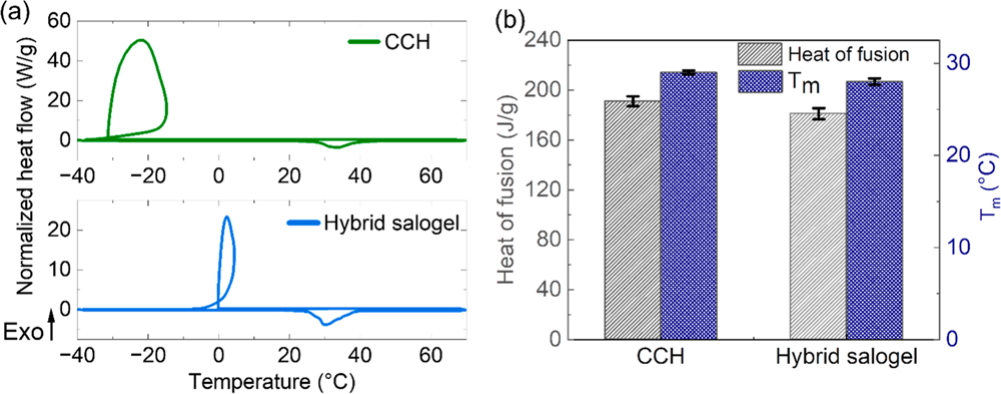
(a) DSC curves for CCH and hybrid (2% PAAm/2% PVA/borax 10 mol %) salogel. (b) Heat of fusion and melting temperature in pristine CCH and hybrid salogels in CCH.

**Figure 6:**
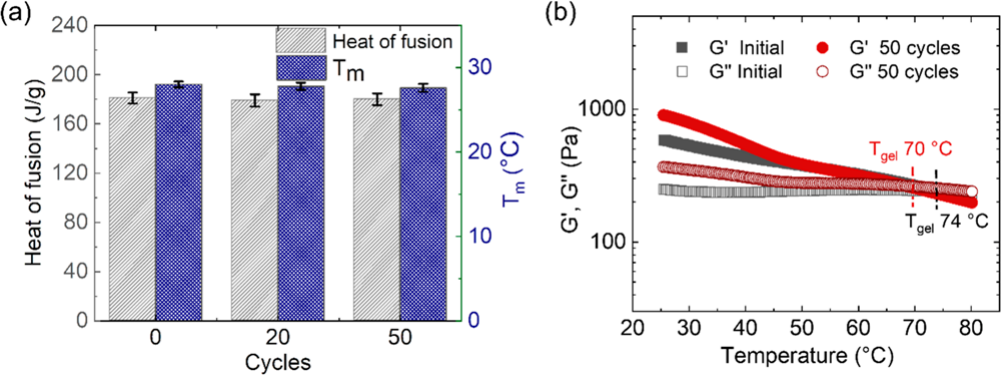
(a) Heat of fusion and melting temperature for hybrid (2% PAAm/2% PVA/borax 10 mol %) salogel and (b) temperature sweep rheology experiments comparing *G*′, *G*′′, and *T*_gel_ of hybrid salogel before and after thermal cycling. Temperature sweep rheology experiments were performed at a frequency of 10 rad/s and 1% strain.

We then aimed to explore the thermal energy storage capability of the hybrid salogels during multiple melting and crystallization cycles that is crucial for long-term use of these materials in thermal energy storage applications. The best-performing salogel system containing PAAm and PVA in equal amounts and the highest borax concentration which could only be achieved in the hybrid system (2% PAAm, 2% PVA, and borax 10 mol %) was chosen for these experiments. Temperature ramps performed using DSC showed that the phase transitions (melting and crystallization) of CCH were intact within the salt hydrate entrapped within the salogel (Figure 5(a)). In the thermograms, crystallization appears as a loop due to the substantial undercooling exhibited by this system and the abrupt release of heat which accompanies nucleation followed by rapid solidification of a metastable liquid. This feature is due, in part, to the high sample mass (9.7 mg) and high scanning rate (10 °C/min). Heat of fusion and melting temperature measurements from DSC experiments revealed that the thermal energy storage capability of the salogel was consistent with the amount of CCH (∼95 wt %) in the salogel (Figure 5(b)), suggesting that the thermal transitions of CCH were not affected by the presence of entanglements, hydrogen bonds, and dynamic covalent crosslinks in the polymer networks. The melting temperature was lowered only by ∼1 °C in the salogel as compared to that of pristine CCH. In contrast, the crystallization temperature was increased by ∼31 °C in the DSC indicating a reduced degree of supercooling in the salogel as compared to that of neat salt hydrate PCM (Figure 5(a)). The thermal properties were also measured after 50 melting and crystallization cycles as described in Materials and Methods. The thermal properties of the salt hydrate were retained after this thermal cycling treatment, showing that the salogel network is robust and does not deteriorate the thermal properties of the PCM (Figure 6(a), Figure S15). Temperature sweep rheology experiments performed with the samples which were subjected to thermal cycling showed that the *T*_gel_ and mechanical properties of the salogel were not significantly changed after 50 cycles (Figure 6(b)). Therefore, we conclude that the hybrid polymer network formed by entanglements, hydrogen bonding, and dynamic covalent crosslinks did not deteriorate the thermal properties of the salt hydrate and was able to withstand at least 50 melting and crystallization cycles of CCH.

**Figure 7:**
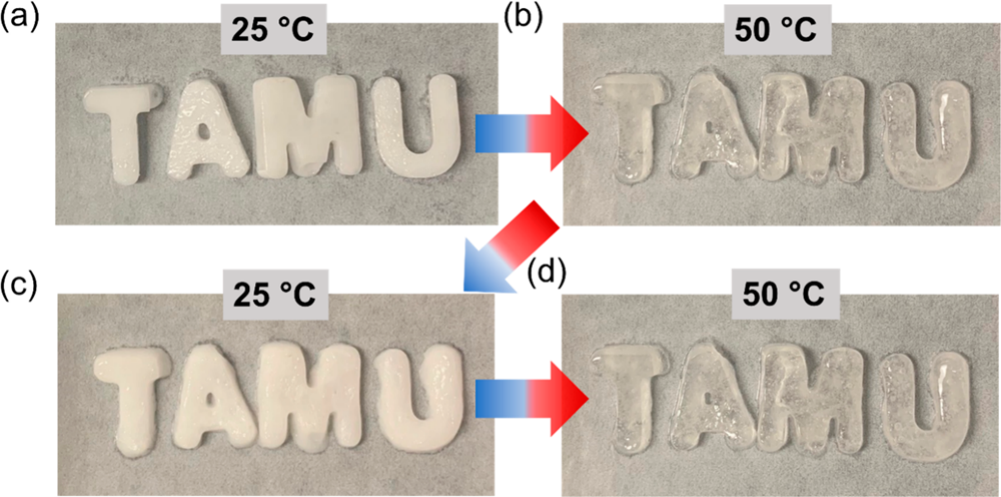
Hybrid salogels (2% PAAm/2% PVA/borax 10 mol %) in “T”, “A”, “M”, and “U” shapes with CCH in (a, c) crystallized state at 25 °C and (b, d) molten state at 50 °C.

Finally, we demonstrate moldability and reprocessing of the hybrid salogels. Figure 7(a) and Figure S16(a) show that that the salogels can be processed in the sol state above their *T*_gel_ by molding them in “T”, “A”, “M”, and “U” shapes and hexagon shapes, followed by crystallizing the salogel-entrapped PCM. In addition, the shape stabilization ability of these salogels was tested by heating the salogel to 50 °C in an airtight container to melt the PCM. The salogel containing PCM in the crystallized state appeared white and turned clear upon melting (Figure 7(a, b) and Figure S16(a, b)). Importantly, no leakage of PCM or loss of shape was observed during melting and after a second crystallization and melting cycle (Figure 7(b–d) and Figure S16(c, d)). These results show that the hybrid polymer matrix enabled the salogel to retain its shape while successfully entrapping CCH.

Table 1 compares hybrid salogels reported in this work with other systems reported in the literature for CCH and its eutectics based on the amount of PCM in the shape-stabilizing matrix and heat of fusion retained. Compared to impregnation of inorganic PCMs in porous matrices,^47−50^ encapsulation in polymer capsules, thickening with cellulosic^23^ and superabsorbent polymer thickeners,^21^ and entrapment in covalently crosslinked PAAm,^40^ the hybrid salogel developed in this work demonstrated more efficient entrapment of the salt hydrate, achieving more than 95% PCM content in the salogel. Due to the ability to trap a large amount of PCM with a small amount of polymer, the hybrid salogels showed the best heat of fusion retention of the pristine salt hydrate PCM. Importantly, the presence of secondary interactions (hydrogen bond, dynamic covalent cross-links) along with entanglements in the hybrid polymer salogels supported shape stability and robust performance during thermal transitions, while conferring on-demand reversibility and tunability of gelation temperature not achievable with individual polymer components.

**Table 1:** Comparison of Developed Salogels with Other Shape Stabilization Systems for Chloride Salt Hydrates Reported in Literature

	Matrix	PCM amount (wt %)	% Heat of fusion retention	Reversibility
Zou et al. (CaCl_2_·6H_2_O)^47^	Expanded graphite	85	56	No
Fu et al. (CaCl_2_·6H_2_O)^48^	Expanded perlite	55	46	No
Zhang et al. (CaCl_2_·6H_2_O)^49^	Diatomite	58.3	57	No
Tan et al. (CaCl_2_·6H_2_O)^50^	Expanded graphite	88	89	No
Wu et al. (CaCl_2_·6H_2_O)^51^	VOOH capsules	N/A	53	No
Yang et al. (CaCl_2_·6H_2_O)^52^	Polysiloxane/polyurea capsules	75	62	No
Bao et al. (CaCl_2_·6H_2_O)^21^	Superabsorbent polymer thickener	75	74	N/A
Li et al. (CaCl_2_·6H_2_O-MgCl_2_·6H_2_O)^23^	Hydroxyethyl cellulose	77	73	N/A
Wang et al. (CaCl_2_·6H_2_O-MgCl_2_·6H_2_O)^40^	Covalently cross-linked PAAm	96.2	75	No
This work (CaCl_2_·6H_2_O)	PAAm/boronate ester hybrid	95.2	95	Yes

## Conclusions

A hybrid salogel design strategy was demonstrated based on a combination of physical entanglements and dynamic covalent cross-links to shape stabilize an inorganic PCM. The strategy was necessitated due to the significant effect of the anion type on gelation behavior of polymers in molten salt hydrates. The target PCM in this work was calcium chloride hexahydrate (CCH), an inexpensive and widely available salt hydrate PCM with a high heat of fusion and near ambient melting temperature. However, weaker PVA/borax gels with lower *T*_gel_ were formed in the chloride salt hydrate CCH in comparison to the previously studied nitrate salt hydrate, CNH, due to the strong salting-out effect of chloride ions. To achieve shape stabilization at low polymer concentration (<5 wt %) with thermo-reversible gelation and tunable *T*_gel_ in CCH, a combination of boronate ester crosslinks with physical entanglements was used in this work by introducing an ultrahigh molecular weight PAAm. Hydrogen bonding between PAAm and PVA within a joint, hybrid network supported a synergistic effect between the entanglements and dynamic covalent crosslinks to yield robust, shape stable yet temperature responsive salogels. The salogels formed using this strategy retained ∼95% of the heat of fusion of CCH and only a small change in melting temperature while also providing shape stabilization above *T*_m_ of CCH and processability above *T*_gel_, all at a low polymer and crosslinker concentration of ∼4.8% that is essential for retention of high efficiency of this thermal energy storage materials. Finally, the hybrid salogels were easily moldable and retained their mechanical and thermal properties after 50 melting/crystallization cycles.

## Supporting Information Available

Salogel preparation flowchart, comparison of properties of thickeners, covalent networks and salogels, FTIR spectra of deuterated and nondeuterated salt hydrates, FTIR spectra of PAAm in CCD and D_2_O, temperature sweep rheology data for PAAm, PVA/borax, and hybrid salogels, tan δ as a function of PAAm and borax concentration at 40 °C, heat of fusion and melting temperature as a function of PAAm concentration, DSC curves for hybrid salogel before and after thermal cycling, vial inversion pictures for PVA in CC*n*H and CN*n*H, PAAm (6000 kDa) in water and PAAm (150 kDa) in CCH, and images showing moldability and shape stabilization (PDF)
