# Supplementary material for: Hybrid Polymer Salogels for Reversible Entrapment of Salt-Hydrate-Based Thermal Energy Storage Materials
Source: ACS Appl Eng Mater. 2023 Dec 8;2(3):553–62. doi: 10.1021/acsaenm.3c00522 (PMC10964238; doi:10.1021/acsaenm.3c00522)
Supplement: Supplementary file 1 — em3c00522_si_001.pdf [file em3c00522_si_001.pdf]

Supporting Information

Hybrid Polymer Salogels for Reversible Entrapment of Salt-Hydrate-Based Thermal Energy Storage Materials

Kartik Kumar Rajagopalan,[1] Sebastian Haney,[2] Patrick J. Shamberger,[1] and Svetlana A. Sukhishvili[1*]

[1] Department of Materials Science & Engineering, Texas A&M University, College Station, TX 77843, USA

[2] Department of Materials Science & Engineering, University of California, Berkeley, CA 94720, USA

*Corresponding author email address: svetlana@tamu.edu

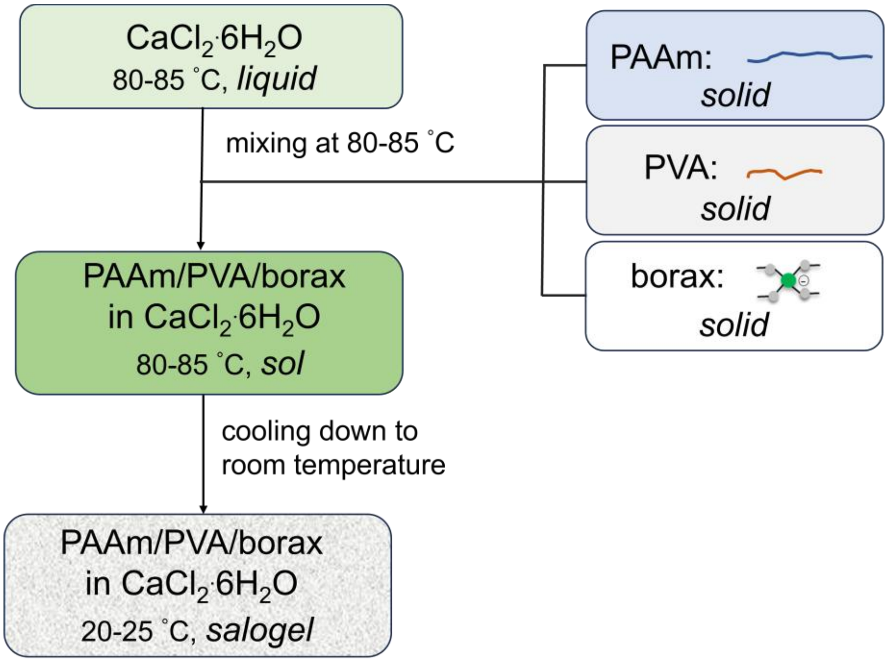

Scheme 1. Flowchart showing steps involved in hybrid salogel preparation.

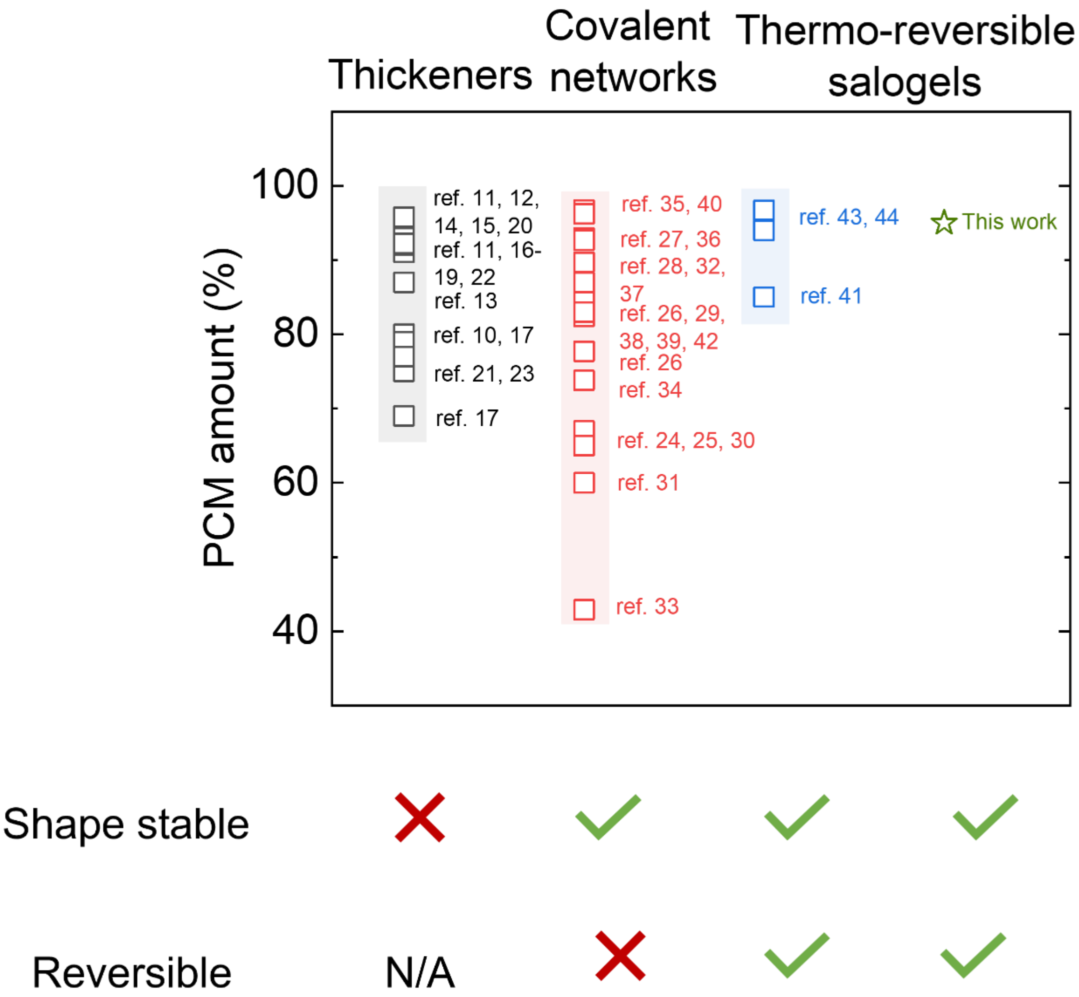

Fig. S1. Comparison of properties of thickeners, covalent networks, and salogels from literature.

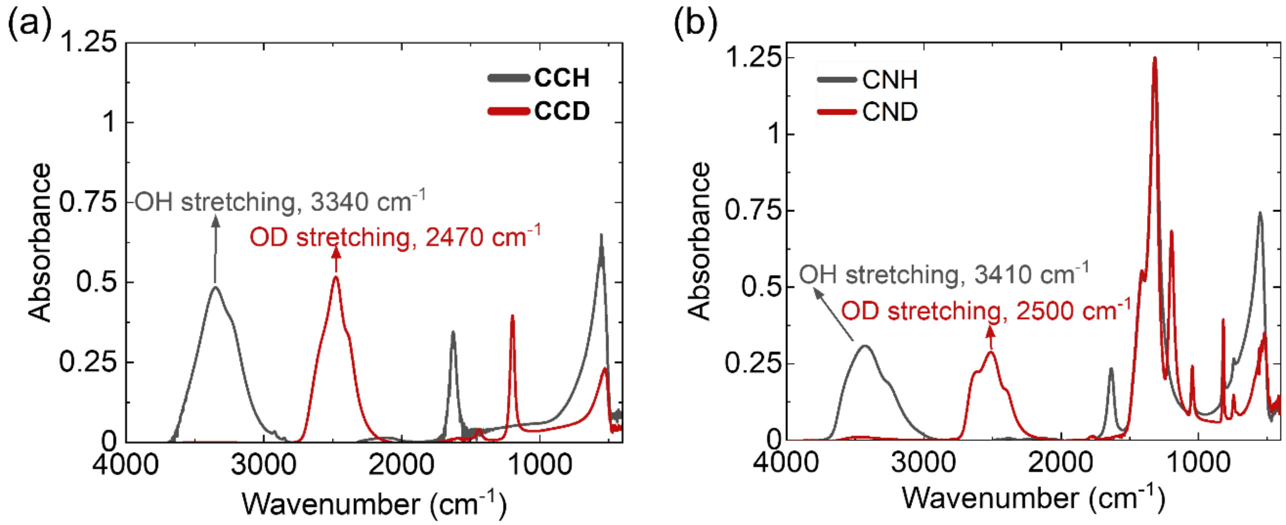

Fig. S2. ATR-FTIR spectra showing the shift of -OH peak of water in CCH and CNH after deuteration to CCD and CND.

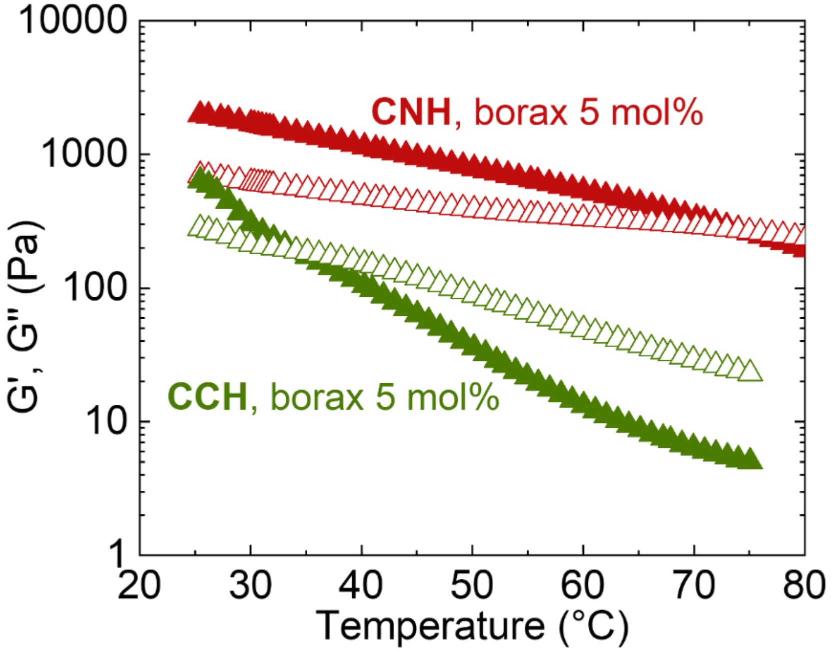

Fig. S3. Oscillatory rheology temperature sweep experiments performed at a frequency of 10 rad/s and 1% strain comparing PVA/borax gels in CNH and CCH. PVA concentration was 3 wt%.

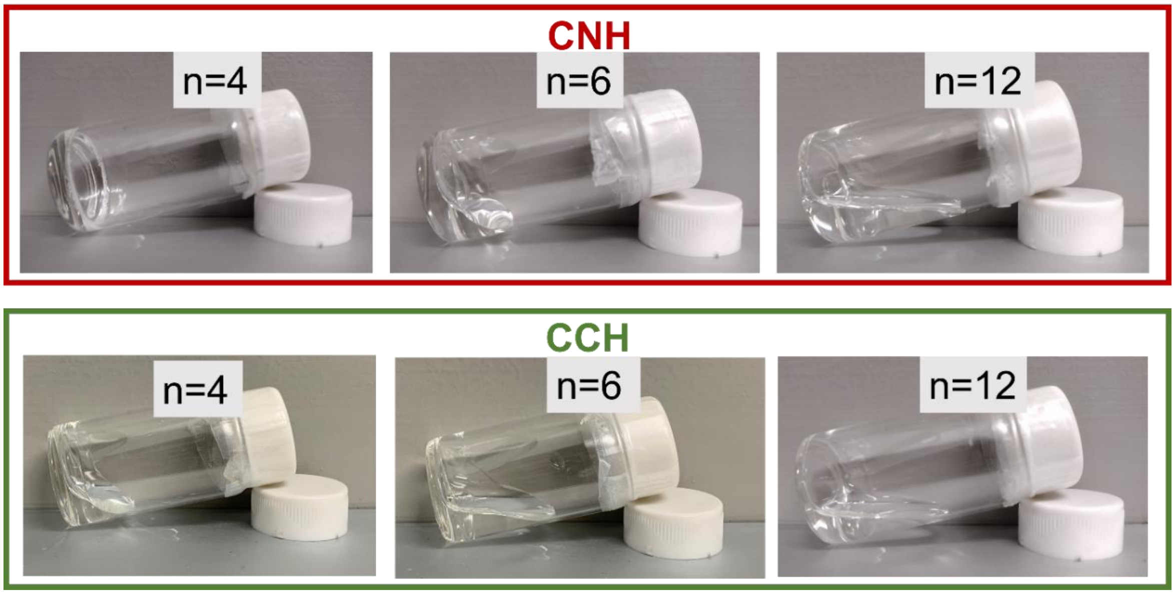

Fig. S4. Vial inversion experiments performed at 25 °C showing the effect of water content on gelation behavior of PVA in CN*n*H and CC*n*H. PVA concentration was 5 wt%.

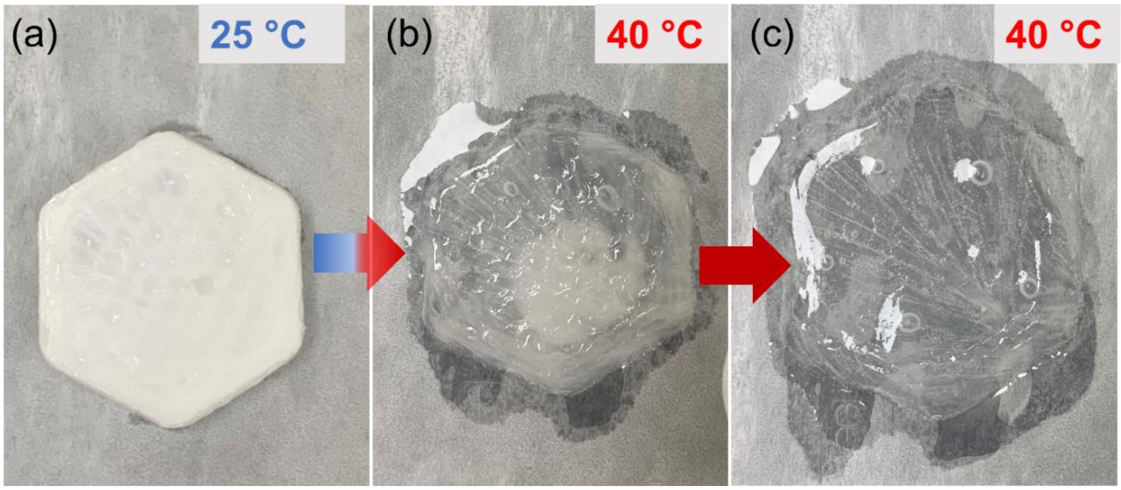

Fig. S5. Leakage of CCH and loss of salogel shape during melting in 3% PVA/5 mol% borax salogels. (a) Salogel with CCH in crystallized state. (b) Leakage of CCH during melting at 40 °C. (c) Leakage of CCH and loss of salogel shape upon complete melting of CCH at 40 °C. Complete melting took about 20 minutes.

3% PAAm 6000 kDa, H_2O

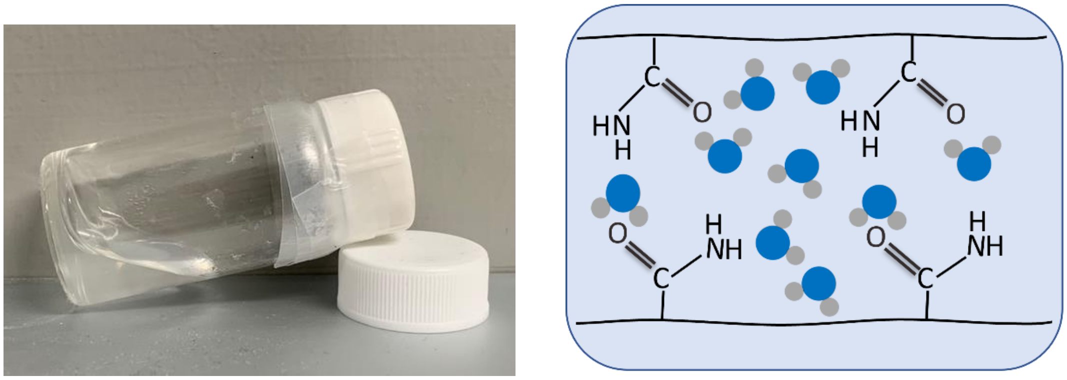

Fig. S6. Lack of gelation of PAAm 6000 kDa in water from vial inversion and schematic showing hydration of amide groups on PAAm backbone.

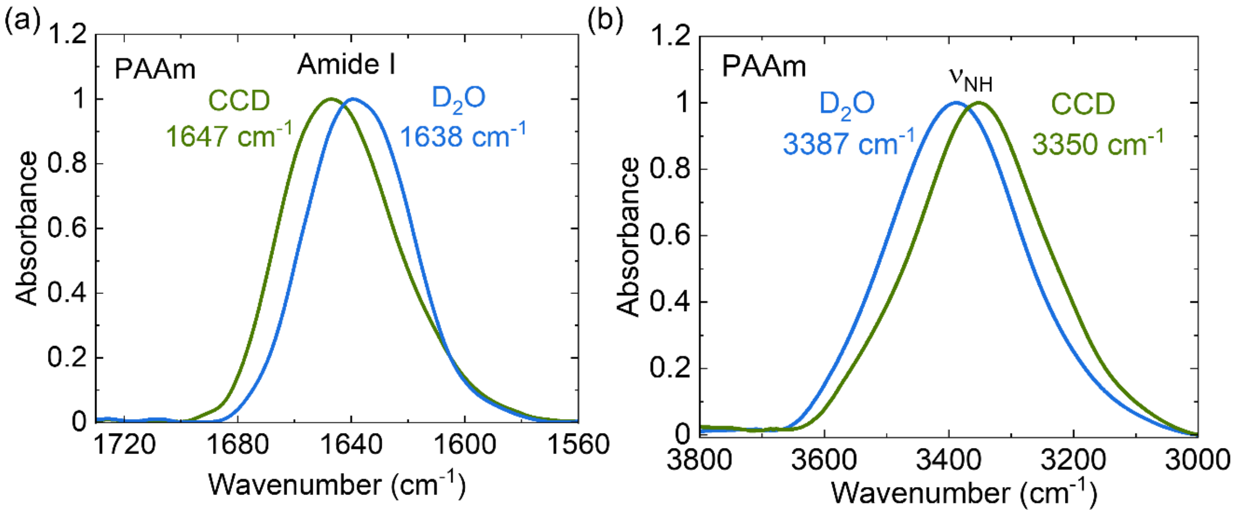

Fig. S7. ATR-FTIR spectra showing changes in the (a) amide I peak and (b) -NH stretching peaks in CCD and D_2O. Polymer concentration was 10 wt%.

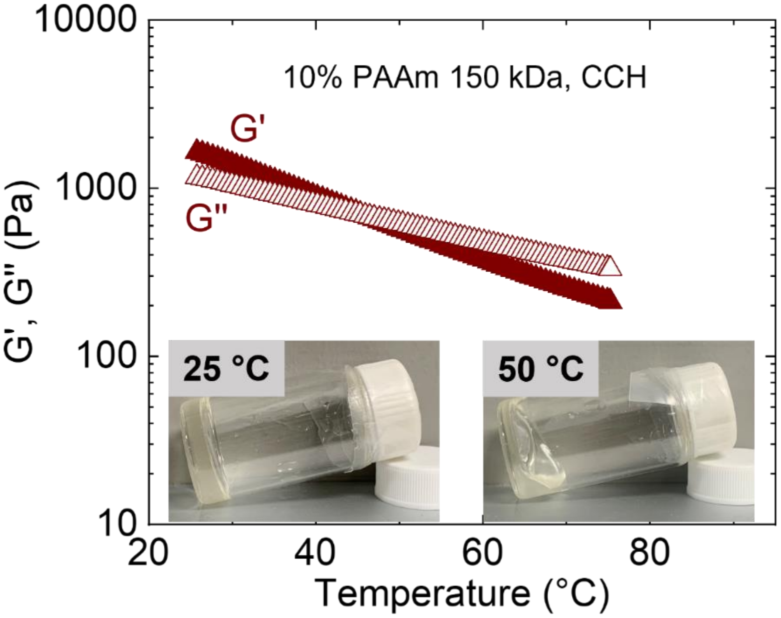

Fig. S8. Temperature sweep rheology plot showing gel-sol transition of 10% PAAm 150 kDa gel in CCH. Inset shows pictures of gel-to-sol transition from vial inversion.

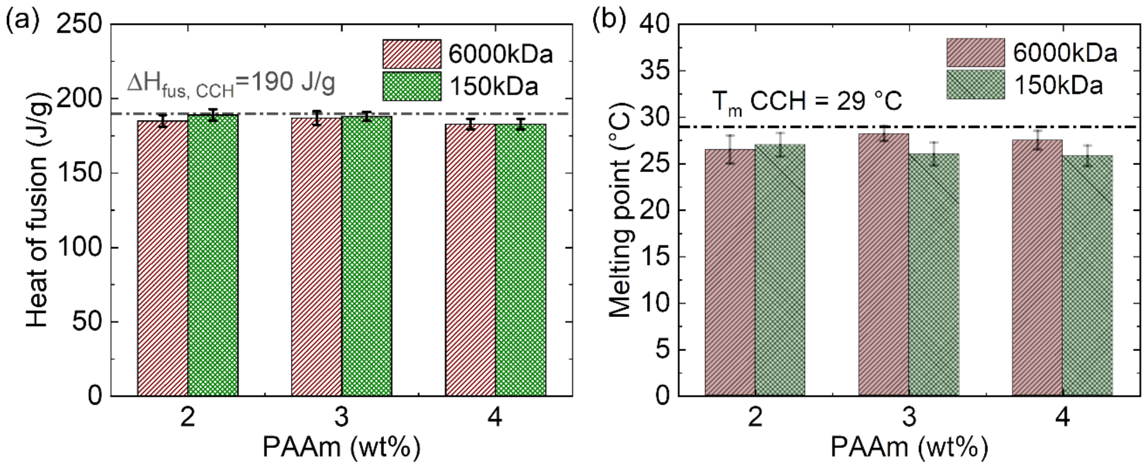

Fig. S9. (a) Heat of fusion and (b) melting temperature as a function of polymer concentration for PAAm (6000 kDa) and PAAm 150 kDa in CCH.

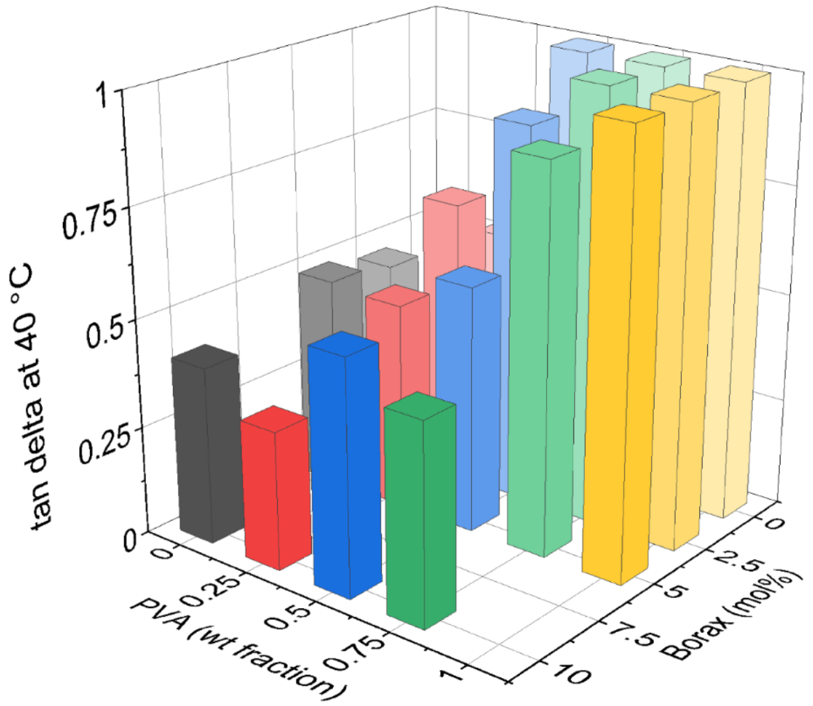

Fig. S10. 3D plot showing *tan δ* at 40 °C obtained from temperature sweep rheology experiments for PAAm, PVA/borax, and hybrid salogels. Note that the borax scale reads from 10 to 0 here to enable easy visibility of the data points with low tan δ values.

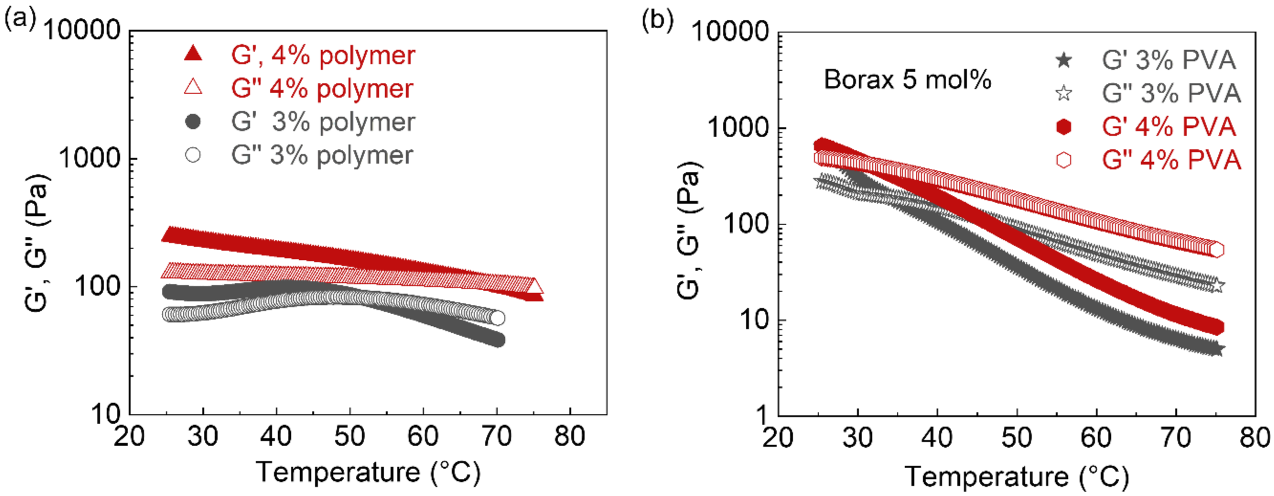

Fig. S11. Temperature sweep rheology plots showing the effect of polymer concentration (3% and 4%) in (a) hybrid salogel (PAAm:PVA 50:50 by weight) and (b) PVA salogels. Borax concentration is 5 mol% in both (a) and (b).

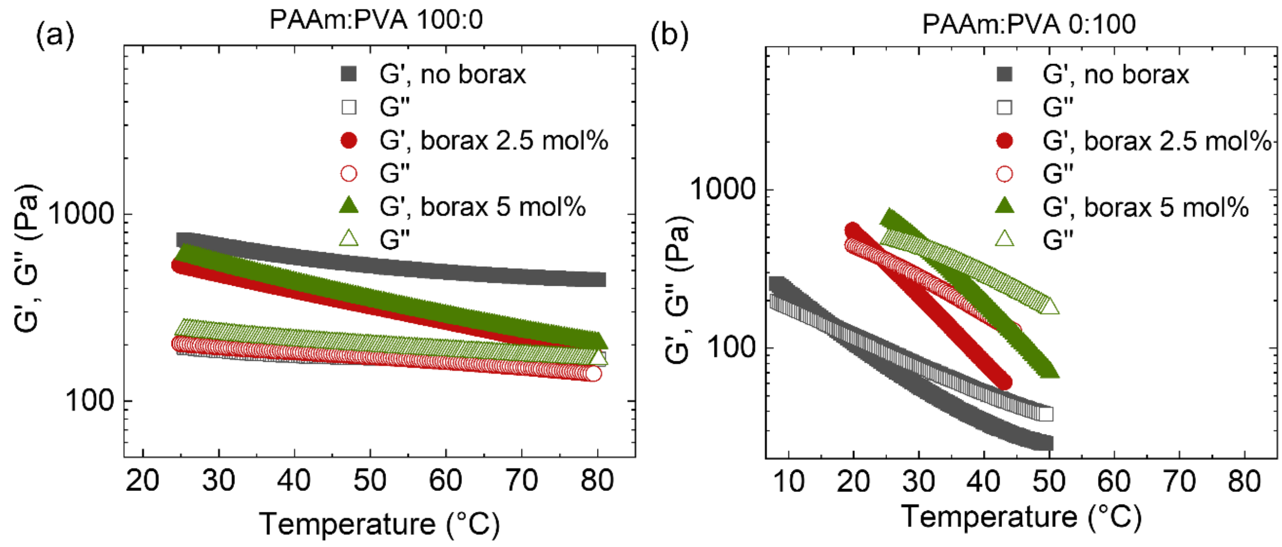

Fig. S12. Temperature sweep rheology plots showing G' and G'' as a function of temperature for different borax concentrations for (a) PAAm salogels and (b) PVA salogels.

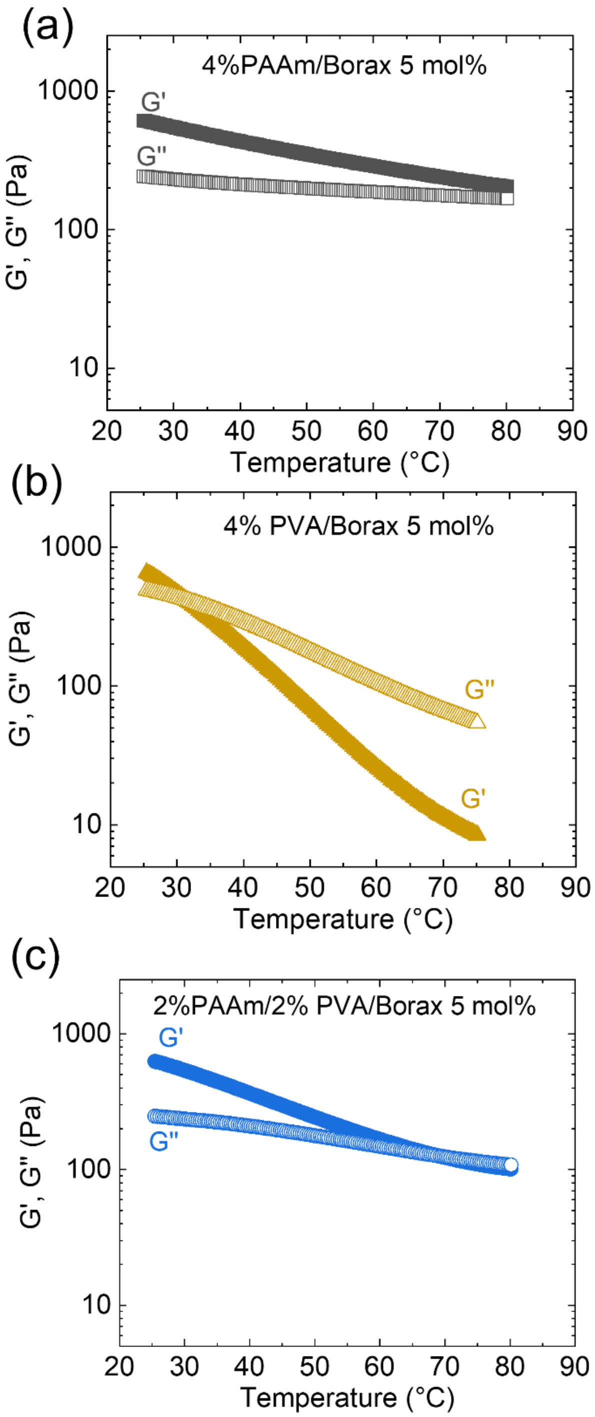

Fig. S13. Temperature sweep rheology plot comparing (a) PAAm, (b) PVA/borax, and (c) PVA/borax/PAAm hybrid salogel at matched total polymer concentration of 4% and borax concentration of 5 mol% to PVA hydroxyl groups.

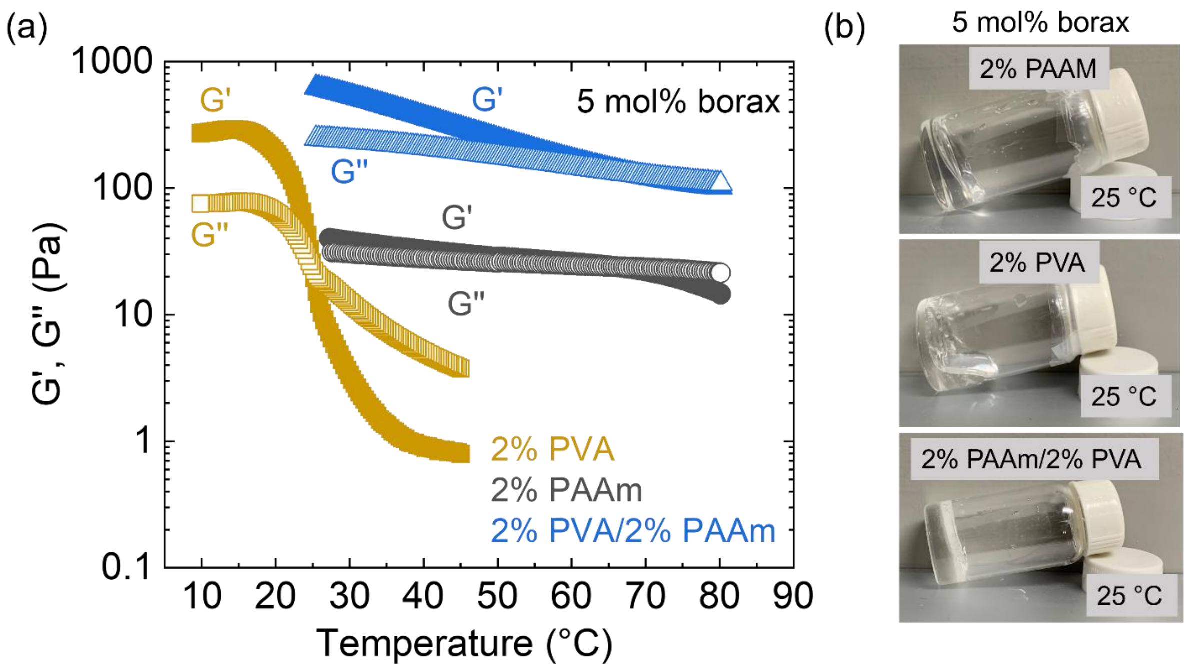

Fig. S14. (a) Temperature sweep rheology plots showing the PAAm, PVA/borax, and PAAm/PVA/borax hybrid salogels. Note that PVA/borax and PAAm salogels contains 2% polymer, and the hybrid salogel contains 4% polymer (equal amounts of PVA and PAAm). (b) Vial inversion experiments showing shape stabilization capability of PAAm, PVA/borax, and hybrid salogel at 25 °C.

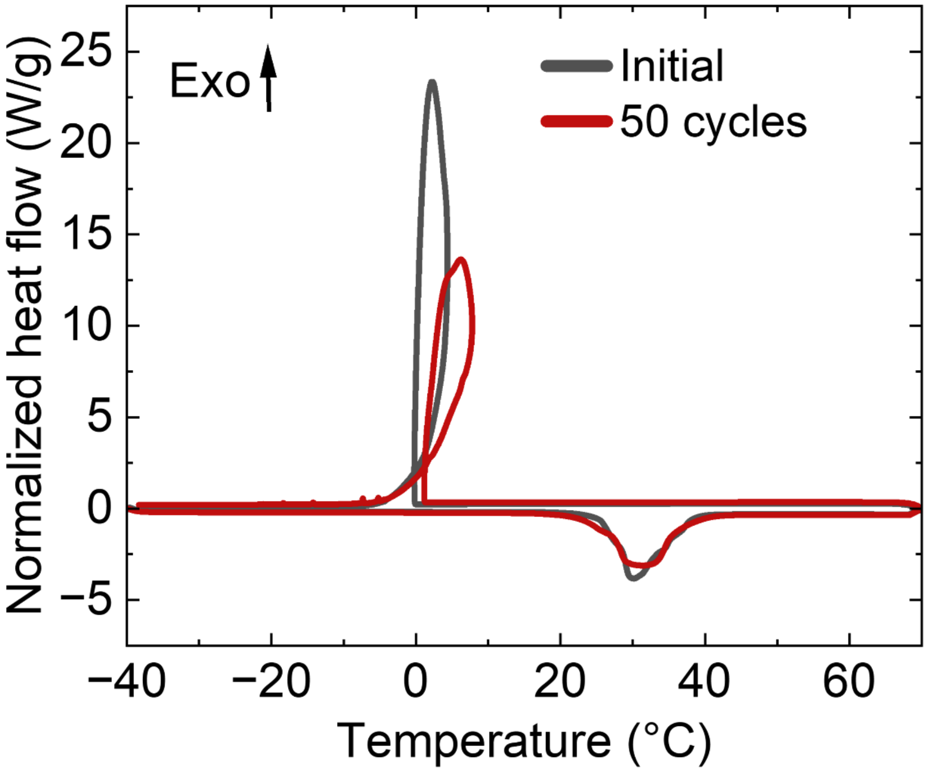

Fig. S15. DSC curves showing melting and crystallization peaks of CCH in 2% PAAm/2% PVA/borax 10 mol% hybrid salogel before and after 50 melting/crystallization cycles.

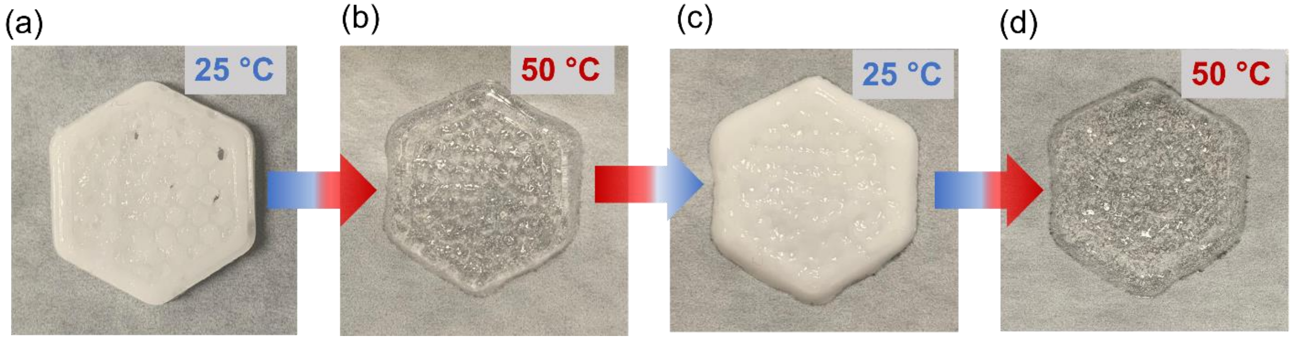

Fig. S16. Hybrid salogel (2% PAAm/2% PVA/borax 10 mol%) in hexagon shape showing shape stabilization and leakage prevention of CCH. (a), (c) Salogel with CCH in crystallized state. (b), (d) Salogel with CCH in melted state.
